# Elicitor-Induced Production of Biomass and Pharmaceutical Phenolic Compounds in Cell Suspension Culture of Date Palm (*Phoenix dactylifera* L.)

**DOI:** 10.3390/molecules25204669

**Published:** 2020-10-13

**Authors:** Jameel Mohammed Al-Khayri, Poornananda Madhava Naik

**Affiliations:** 1 Department of Agricultural Biotechnology, College of Agriculture and Food Sciences, King Faisal University, P.O. Box 420, Al-Ahsa 31982, Saudi Arabia; 2 Department of Botany, Karnatak University, Dharwad 580003, India

**Keywords:** antioxidant activity, biomass, cell culture, elicitor, date palm, flavonoid, phenolic compounds, salicylic acid

## Abstract

Plants that synthesize bioactive compounds that have high antioxidant value and elicitation offer a reliable in vitro technique to produce important nutraceutical compounds. The objective of this study is to promote the biosynthesis of these phenolic compounds on a large scale using elicitors in date palm cell suspension culture. Elicitors such as pectin, yeast extract (YE), salicylic acid (SA), cadmium chloride (CdCl_2_), and silver nitrate (AgNO_3_) at 50, 100, and 200 mg/L concentrations are used. The effects of elicitors on cell culture were determined in terms of biomass [packed cell volume (PCV), fresh and dry weight], antioxidant activity, and phenolic compounds (catechin, caffeic acid, kaempferol, apigenin) were determined using high-performance liquid chromatography (HPLC). Results revealed that enhanced PCV (12.3%), total phenolic content [317.9 ± 28.7 mg gallic acid equivalents (GAE)/100 g of dry weight (DW)], and radical scavenging activity (86.0 ± 4.5%) were obtained in the 50 mg/L SA treated cell culture of Murashige and Skoog (MS) medium. The accumulation of optimum catechin (26.6 ± 1.3 µg/g DW), caffeic acid (31.4 ± 3.8 µg/g DW), and kaempferol (13.6 ± 1.6 µg/g DW) was found in the 50 mg/L SA-treated culture when compared to the control. These outcomes could be of great importance in the nutraceutical and agronomic industries.

Academic Editor: Federica Pellati, Laura Mercolini and Roccaldo Sardella

## 1. Introduction

The date palm (*Phoenix dactylifera* L.) is a desert fruit tree known as the “tree of life” for its many uses; it is mainly distributed in Middle Eastern and North African countries [1]. Date fruits are considered as sacred and are good sources of essential dietary minerals with important medicinal and nutritional values [1,2]. These fruits possess a wide range of phenolic compounds with antioxidant activity [3]. Phenolic compounds such as catechin, caffeic acid, kaempferol, and apigenin possess purported anticancer, antidiabetic, antiaging, antiviral, and antimicrobial properties; also, they are used to treat neurodegenerative and cardiovascular diseases and are beneficial to human health [2,4,5].

Plants synthesize bioactive compounds naturally as a defense mechanism in response to pathogen attacks. The same nature and response have been found in plants when treated with chemicals of pathogenic origin (elicitors). Elicitors are stress inducers that stimulate secondary pathways resulting in the synthesis of bioactive compounds. Abiotic and biotic elicitors were employed in vitro to induce a large quantity of bioactive compounds (alkaloids, alkamides, glucosinolates, terpenes, saponins, flavonoids, steroids, phenolics, and coumarins) within a short period of time [6,7,8]. Elicitors of fungal, bacterial, and yeast extract (YE), heavy metals, and hormonal origin were studied for various bioactive compound synthesis [6,9,10,11]. In vitro cultured plant cells such as callus or cell suspension cultures have the ability to produce industrially useful bioactive compounds with great antioxidant activity [5,12]. The application of these elicitors in cell suspension culture will boost the accumulation of phenolic compounds.

The current study evaluated the effects of various elicitors such as pectin, YE, salicylic acid (SA), cadmium chloride (CdCl_2_), and silver nitrate (AgNO_3_) on the packed cell volume (PCV), fresh weight (FW) and dry weight (DW), total phenol and flavonoid contents, antioxidant activity, and production of catechin, caffeic acid, kaempferol, and apigenin in cell suspension culture of the date palm. To our knowledge, this is the first study of the effect of elicitors on the cell suspension culture of date palm. The present results will contribute to a reliable protocol for a date palm cell culture system to develop the bioreactor-scale production of bioactive compounds.

## 2. Results

### 2.1. Influence of Elicitors on Biomass Accumulation

**Figure 1:**
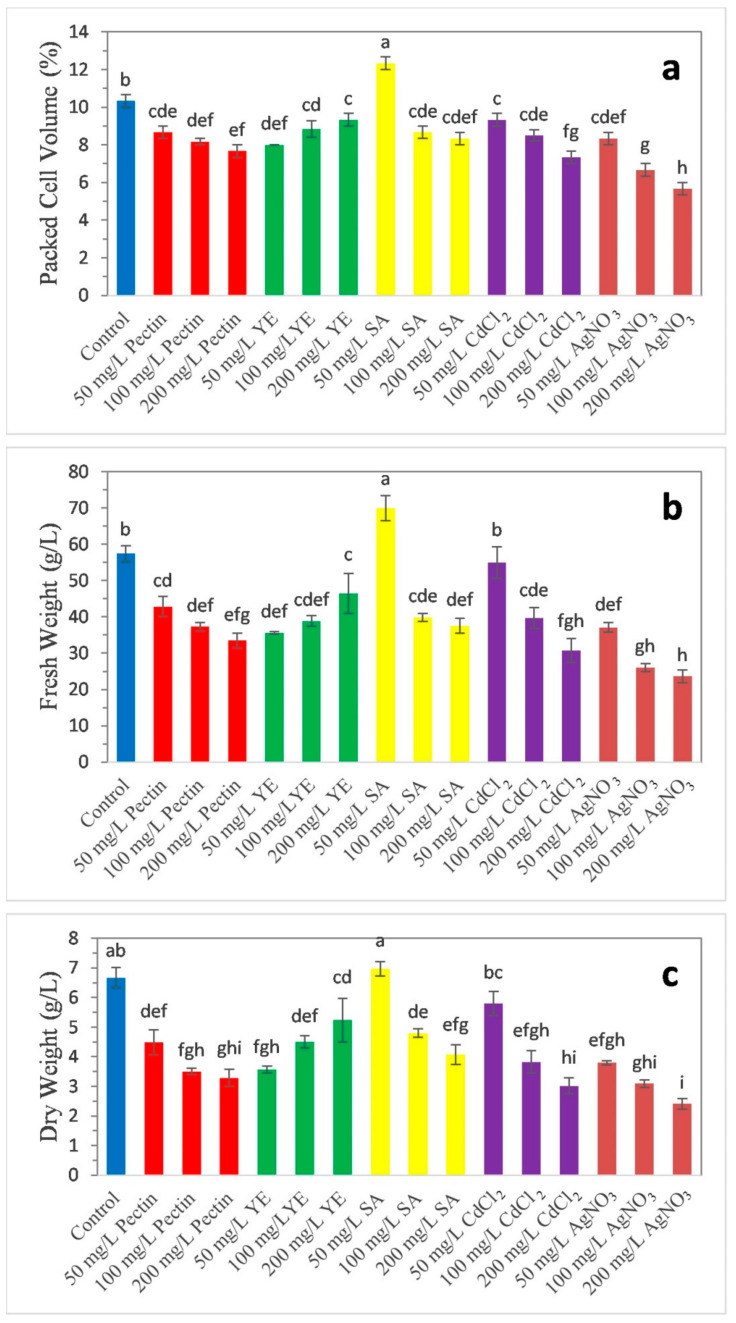
Effect of elicitors on biomass accumulation in date palm cell suspension culture: (**a**) packed cell volume (%), (**b**) fresh weight (g/L), (**c**) dry weight (g/L). Values are represented in means of triplicate with the standard error. Mean values following the same letters in columns are not significantly different, according to Duncan’s multiple range (*p* ≤ 0.05) test.

Cell suspension cultures of date palm treated with various elicitors such as pectin, SA, CdCl_2_, and AgNO_3_ (50, 100, and 150 mg/L concentrations each elicitor) showed decreased PCV, FW, and DW as the concentration increased, except for the YE-treated cell culture, whereas PCV, FW, and DW accumulation decreased as the concentration of the elicitor increased (Figure 1). Among the elicitors and control tested for PCV, FW, and DW accumulation in the cell suspension culture, 50 mg/L SA treatment emerged as a promising dose to induce PCV (12.33%), FW (69.960 ± 3.420 g/L), and DW (6.973 ± 0.237 g/L). The least production of PCV (5.66%), FW (23.653 ± 1.771 g/L), and DW (2.406 ± 0.185 g/L) was observed in 200 mg/L AgNO_3_ treated cell culture.

### 2.2. The Effect of Elicitors on Total Phenolic Content (TPC), Total Flavonoid Content (TFC), and Percentage of Radical Scavenging Activity (RSA)

Date palm cell culture extract was estimated spectrophotometrically, and the results are presented in Table 1. The significant decrease in the phenolic and flavonoid content was observed at the different concentrations of pectin-treated cell culture extract. As the concentration increased, content decreased. The combined accumulation of phenolic and flavonoid content also affects the percentage of radical scavenging activity (%RSA), in which it decreased as the concentration increased. In the YE-treated cell extract, there was increased total phenolic content (TPC) observed as the concentration increased, and at 200 mg/L YE treatment, it reached a maximum of 234.696 ± 36.761 mg gallic acid equivalents (GAE)/100 g of DW, but this quantity was lower than that of the control treatment (266.750 ± 8.746 mg GAE/100 g of DW). Significantly increased TPC (317.986 ± 28.743 mg GAE/100 g of DW) and total flavonoid content (TFC, 157.286 ± 20.775 mg QE/100 g of DW) was obtained in the cell culture treated with a low concentration of elicitors 50 mg/L SA and 50 mg/L CdCl_2_, respectively, when compared to the control experiment. The 100 mg/L CdCl_2_ treatment induced the lowest accumulation of TFC in cell culture extract when compared to all other elicitor treatments and controls. In the AgNO_3_ experiment, higher concentrations inhibit the accumulation of TPC, TFC, and %RSA in cell culture extract. No significant difference of %RSA was seen in the control, 50 mg/L pectin, 50 mg/L SA, and 50 mg/L CdCl_2_ treated cell culture extracts.

### 2.3. Impact of Elicitors on Phenolic Compounds Production

From the various elicitors such as pectin, SA, CdCl_2_, and AgNO_3_ (50, 100 and 200 mg/L) along with control, the culture showed a steady decline in the phenolic content from low concentration to high, except in YE-treated culture (Table 2). The YE-treated cell culture showed increased phenolic content with respect to the increased concentrations, but an accumulation of phenolic compounds was significantly lower than the control culture. The optimum accumulation of catechin (26.6 µg/g DW), caffeic acid (31.4 µg/g DW), and kaempferol (13.6 µg/g DW) was observed at 50 mg/L SA-treated cell culture when compared to the control. Apigenin accumulation was found at a maximum in the control culture. The AgNO_3_ supplemented cell cultures accumulated, significantly, the lowest phenolic content among the tested elicitors.

## 3. Discussion

### 3.1. Biomass Accumulation

The biomass of the cell is the one of the most important factors to measure growth. Different concentrations of elicitor play an important role in cell growth. However, a higher concentration of elicitors induces hypersensitive response, leading to cell death, and an optimum level of elicitor was required for the induction [6]. Our results corroborate the studies of Cai et al. [13], where the *Changium smyrnioides* suspension cells treated with different elicitors [methyl jasmonate (MeJA), SA, calcium chloride (CaCl_2_), copper sulfates (CuSO_4_) and AgNO_3_] at all tested concentrations showed a negative impact on biomass accumulation, and higher concentrations suppressed cell growth significantly. Similar to our results, *Ocimum bacilicum* cell suspension cultures treated with different concentrations of CdCl_2_ and AgNO_3_ ended up with decreased cell dry weight as the concentration of the elicitors increased [14]. The prolonged incubation period and increased elicitor concentration affect the cultures viability [15]. In *Mentha piperita* suspension cultures, biomass growth was inhibited slightly due to the higher concentrations (100 and 200 µM) of jasmonic acid (JA) [16].

### 3.2. Total Phenolic, Flavonoid Content, and %RSA

In the phenylpropanoid pathway, phenolics and flavonoids are synthesized, triggering the key enzymatic pathway, resulting in the accumulation of bioactive compounds [17]. Similar to our findings, Ali et al. [18] reported that the elicitor with a low concentration enhanced the TPC and TFC production in cell suspension culture of *Artemisia absinthium*, and higher concentrations inhibited the production. Callus cultures of *Zingiber officinale* treated with 50 mg/L SA significantly induced a high amount of TPC [11]. The cell culture of *Orostachys cartilaginous* treated with 100 μM SA also showed an optimum production of TPC and TFC [19]. In agreement with our results, *Ocimum bacilicum* cell suspension cultures treated with 200 mg/L YE showed the highest TPC and TFC [14]. Contrary to our findings, El-Nabarawy et al. [20] in their study reported a significant increase in TPC when the *Zingiber officinale* callus culture medium was augmented with a low concentration of YE; meanwhile, a higher concentration of YE did not facilitate the phenolic synthesis. In regenerated shoot cultures of *Salvia virgata*, enhanced TPC and TFC production was observed when treated with a low concentration of Ag^+^ ions, MeJA and YE [21]. 

A direct correlation between TPC, TFC, and the antioxidant activity of date fruits was also recorded [22]. In the elicitor experiment, *Artemisia absinthium* cell suspension culture treated with 1.0 mg/L of MeJA, JA, and gibberellic acid (GA) each recorded the highest RSA [18]. The lower (0.5 mg/L) and higher concentrations (2 mg/L) of jasmonates and GA treatments resulted in the inhibition of RSA. The metabolic profiles of 18 Saudi date palm fruit cultivars were examined by Farag et al. [3] and revealed that the antioxidant activity mainly depends on the combined effect of the total compounds, rather than individual phenolics. In the present study, cumulative TPC and TFC values determine the RSA in the cell culture extract of date palm. This indicates that in cell cultures of date palm, the production of nutraceutically active compounds is possible.

### 3.3. Production of Bioactive Compounds

Elicitation is one of the strategies employed in plant cell culture to improve the productivity of bioactive compounds [23]. Various parameters such as the nature of the elicitor, concentrations, and duration of elicitor exposure are important factors to induce the optimum level of bioactive compounds [6]. Baldi et al. [24] studied the effect of various abiotic elicitors viz. arachidonic acid, MeJA, CaCl_2_, and CuSO_4_ and biotic elicitors viz. *Alternia alternate*, *Fusarium solani*, and *Verticilium dahaliae* on the production of withaferin A from transformed cell cultures of *Withania somnifera* and found 5.4 and 9.7 times higher production, respectively, with copper sulfate (100 μM) and the cell extract of *Verticilium dahaliae* (5% *v/v*). The dual elicitation strategy by the combined addition of these two elicitors resulted in a 13.8-fold enhancement of withaferin A content in comparison to control cultures. In plants, heavy metals can bring changes in metabolic activity, which affects the photosynthetic pigments, proteins, and sugars. These effects are because of the involvement of the inhibition of enzymes in the production of bioactive compounds [6]. Cai et al. [13] reported the elicitation of furanocoumarins in *Changium smyrnioides* suspension cells, where elicitors such as MeJA, SA, and CaCl_2_ triggered furanocoumarin accumulation and suppressed cell growth, while heavy metals such as CuSO_4_ and AgNO_3_ showed little effect. Contrary to our findings, *Ocimum bacilicum* cell cultures treated with YE accumulated the highest chicoric acid and rosmarinic acid when compared to the control [14]. The concentrations of SA from 25 to 150 μM enhanced the bioactive compound accumulation in cell culture of *Orostachys cartilaginous* without any reduction in the cell biomass [19]. In cell suspension culture of *Salvia miltiorrhiza,* SA as an elicitor significantly increased the biosynthesis of 10 bioactive compounds at different times [25]. In agreement with our results, the *Bacopa monnieri* suspension cultures treated with SA induced the maximum biomass and bacoside content [26].

## 4. Materials and Methods

### 4.1. Plant Material

Date palm cv. Shishi was used in this study. It is an inexpensive and abundantly available date cultivar of the Al-Ahsa region, Saudi Arabia, where this study was conducted. The tissue culture protocol for date palm developed by Al-Khayri [27], Naik, and Al-Khayri [28] was followed for explant preparation and friable callus induction.

### 4.2. Cell Suspension Culture

The scalpel-macerated friable calli (0.5 g per flask) were inoculated in 250-mL conical flasks containing 50 mL MS [29] liquid medium to induce cell cultures (MS media chemicals and growth hormones are obtained from HiMedia, Mumbai, India, PTC grade). The medium was supplemented with MS salts and a hormone combination of 1.5 mg/L 2-iP and 10 mg/L NAA, which were treated with different concentrations of individual elicitors. Various elicitors were used along with one set of controls. The tested elicitors (HiMedia, Mumbai, India) were pectin (PTC grade), YE (Technical grade), SA (AR grade), CdCl_2_ (AR grade), and AgNO_3_ (AR grade) at 50, 100, and 200 mg/L concentrations. Cell suspension cultures were incubated in a rotary shaker at 150 rpm and provided a light (40 µmol/m^2^/s) source for 16 h and temperature maintained at 25 ± 2 °C. The cell cultures were maintained up to 11 weeks to achieve maximum growth.

### 4.3. Biomass

PCV is the single method used to determine the cell growth. To estimate PCV, from 11-week-old cell suspension, 10 mL of cell culture was transferred to 15-mL centrifuge tubes (sterile and graduated) and centrifuged for 5 min at 2000× *g*. The PCV was recorded as the cell mass percentage of the total centrifuged volume. The 11-week-old cell cultures were used to determine the FW and DW following the procedure used by Naik and Al-Khayri [5].

### 4.4. Extraction of Cell Culture

The extraction of cell culture was done by using a 100 mg finely powdered sample in a 15-mL centrifuge tube containing 10 mL of 80% methanol (Rankem, Gurgaon, India, HPLC grade) incubated at 60 °C under a water bath for 2 h, as described by Naik and Al-Khayri [5]. The extract was centrifuged at 6000 rpm for 20 min. The supernatant sample was filtered through a 0.45 µm polyvinylidene fluoride (PVDF) membrane filter (Merck, Millipore, Cork, Ireland) and collected in a 125 mL round-bottom flask. This filtrate was dried at 50 °C at 250 rpm using rotary evaporator (OSB-2100 Eyela, Tokyo, Japan). The dried extracts were dissolved in 1 mL of 80% methanol and filtered through a 0.45 µm PVDF membrane filter. The filtrates were collected in 2 mL sample vials.

### 4.5. Total Phenolic and Flavonoid Content

The Folin–Ciocalteu method [30] was used to determine the TPC from date palm cell suspension culture, with some modifications [31]. Methanolic extract (20 µL) was mixed with 1.58 mL of deionized water and 100 µL of Folin–Ciocalteu reagent (SRL, Mumbai, India, AR grade); then, it was allowed to stand for 8 min before 20% of 300 µL sodium carbonate solution (HiMedia, Mumbai, India, AR grade) was added. After shaking and mixing the solution well, we left it for 2 h at 20 °C, after which the color was developed and the absorbance was measured at 765 nm using a UV-visible spectrophotometer (Shanghai Yoke, Shanghai, China). The TPC of cell extract was expressed as mg GAE/100 g of DW. The colorimetric method as explained by Thiruvengadam et al. [32] was used to determine the TFC of date palm cell suspension culture extracts, with some modifications [31]. Methanolic extracts (100 µL), 10% of 50 µL aluminum chloride (SRL, Mumbai, India, AR grade), 1 M of 50 µL potassium acetate (HiMedia, Mumbai, India, AR grade), and distilled water (1.8 mL) were mixed. After incubation for 30 min at room temperature, the absorbance was measured at 415 nm using a UV-visible spectrophotometer (Shanghai Yoke, Shanghai, China). The TFC of cell extract was expressed as mg QE/100 g of DW.

### 4.6. Determination of Radical Scavenging Activity

The stable free radical 2,2-diphenyl-1-picrylhydrazyl [DPPH, (SRL, Mumbai, India, AR grade)] was used to test the antioxidant activities of a particular compound/cell extract due to its hydrogen ion-donating ability [33]. The antioxidant activity of date palm cell extracts was determined by referring to the Farag et al. [3] protocol with some modifications [31]. The original cell extract (sample) in methanol made a stock value of 1 mg/mL. From this stock solution, the sample was prepared to contain different concentrations: 2, 5, 10, 20, 50, 100, 250, 500, and 1000 µg/mL. In the same way as the samples, a standard control butylated hydroxyanisole (SRL, Mumbai, India, AR grade) was prepared. From the original sample extracts, 250 µL were taken to make up the volume to 1 mL with the same solvent. Then, 2 mL of DPPH was added to each test. Finally, a solution containing only 1 mL of methanol and 2 mL of DPPH solution was prepared and used as a blank. All test tubes were incubated in the dark for 30 min at room temperature. The UV-visible spectrophotometer was set at 517 nm and the absorbance was adjusted at zero for methanol. The absorbance of blank, standard control, and samples was recorded.

### 4.7. Chromatographic Analysis of Phenolic Compounds

Chromatographic analysis and the quantification of phenolic compounds was performed by the HPLC system (Shimadzu Prominence Liquid chromatography, Kyoto, Japan), following the procedure used by the Naik and Al-Khayri [5]. Solutions of pure known compounds catechin, caffeic acid, kaempferol, and apigenin (Sigma-Aldrich, St. Louis, MO, USA, HPLC grade) were chromatographed as external standards. The phenolic compounds of cell suspension extracts were identified by comparing their retention times with those of pure standards. Based on the calibration curves of the individual standards, the quantitative data of the sample extracts were calculated. The quantified results of catechin, caffeic acid, kaempferol, and apigenin were indicated as µg/g DW. 

### 4.8. Statistical Analysis

Each treatment consisted of three replicates. Results were evaluated by SPSS statistics version 22.0 (IBM Corp. New York, NY, USA), using analysis of variance (ANOVA). The mean separation was accomplished according to Duncan’s multiple range test at *p* ≤ 0.05 levels.

## 5. Conclusions

The current experiment explains the various elicitor effects on the production of biomass, TPC, TFC, antioxidant activity, and phenolic compounds production from the cell culture of date palm cv. Shishi. The lower concentration of SA favored biomass production. All the treatments of elicitors affect the accumulation of TPC and TFC in cell culture. Furthermore, the accumulation of TPC and TFC showed a positive correlation with antioxidant activity in cell culture of date palm treated with 50 mg/L SA and 50 mg/L CdCl_2_, respectively. The production of phenolic compounds such as catechin, caffeic acid, and kaempferol are also enhanced by treating the cell culture with 50 mg/L SA; these compounds possess possible anticancer, antiaging, antiviral, antimicrobial, and very high antioxidant activity. An overall finding indicates that a low concentration of SA treatment favors the production of biomass, TPC, TFC, RSA, and phenolic compounds. This key information can be utilized for the scale-up production of important bioactive nutraceutical compounds. 

## Figures and Tables

**Table 1:** Effect of elicitors on the accumulation of total phenolic, flavonoid content, and radical scavenging activity (%) in date palm cell suspension culture *.

Elicitors	Total Phenolic Content (mg GAE/100 g DW)	Total Flavonoid Content (mg QE/100 g DW)	Radical Scavenging Activity (%)
Control	266.750 ± 8.746 ab	68.660 ± 12.222 bc	89.86 ± 1.57 a
50 mg/L Pectin	256.910 ± 23.733 ab	74.650 ± 9.530 b	79.75 ± 6.03 a
100 mg/L Pectin	124.656 ± 3.085 def	44.300 ± 1.322 cde	20.85 ± 1.09 ef
200 mg/L Pectin	127.120 ± 17.031 def	33.760 ± 2.592 def	9.700 ± 0.22 f
50 mg/L YE	209.226 ± 1.024 bcd	67.453 ± 2.572 bc	46.66 ± 7.83 cd
100 mg/L YE	215.883 ± 67.611 bc	65.676 ± 13.691 bc	59.59 ± 2.56 b
200 mg/L YE	234.696 ± 36.761 b	72.403 ± 6.671 b	58.49 ± 10.40 bc
50 mg/L SA	317.986 ± 28.743 a	47.340 ± 3.412 cd	86.09 ± 4.54 a
100 mg/L SA	138.566 ± 22.231 cdef	4.056 ± 1.423 g	27.42 ± 1.74 e
200 mg/L SA	129.000 ± 19.555 def	13.120 ± 1.016 fg	22.07 ± 2.97 ef
50 mg/L CdCl_2_	242.620 ± 24.835 ab	157.286 ± 20.775 a	85.25 ± 2.27 a
100 mg/L CdCl_2_	140.613 ± 34.443 cde	0.163 ± 0.026 g	40.83 ± 3.42 d
200 mg/L CdCl_2_	53.766 ± 11.804 f	4.053 ± 0.718 g	21.10 ± 3.24 ef
50 mg/L AgNO_3_	135.716 ± 3.975 cdef	20.940 ± 5.328 efg	63.21 ± 3.01 b
100 mg/L AgNO_3_	55.336 ± 13.768 ef	13.896 ± 2.376 fg	19.59 ± 1.49 ef
200 mg/L AgNO_3_	59.956 ± 7.156 ef	14.260 ± 0.508 fg	19.66 ± 2.09 ef

* Data were collected at the 11th week of culture. Values represent the mean ± SE. Mean values following the same letter within columns are not significantly different, according to Duncan’s multiple range (*p* ≤ 0.05) test.

**Table 2:** Effect of elicitors on the accumulation of phenolic compounds in date palm cell suspension culture *.

Elicitors	Catechin µg/g DW	Caffeic Acid µg/g DW	Kaempferol µg/g DW	Apigenin µg/g DW
Control	21.8 ± 0.5 b	23.2 ± 2.4 b	13.2 ± 0.7 a	30.4 ± 5.7 a
50 mg/L Pectin	17.9 ± 0.7 cd	15.2 ± 0.9 cd	6.1 ± 0.6 cde	25.4 ± 1.4 abc
100 mg/L Pectin	6.3 ± 1.3 f	6.4 ± 2.1 efgh	0.0 ± 0.0 f	23.8 ± 2.0 abc
200 mg/L Pectin	5.5 ± 1.4 f	5.6 ± 1.6 fgh	0.0 ± 0.0 f	26.8 ± 3.8 ab
50 mg/L YE	11.2 ± 1.4 e	10.6 ± 0.7 def	7.1 ± 0.3 cd	22.7 ± 2.9 abc
100 mg/L YE	16.2 ± 1.0 cd	14.4 ± 2.2 cd	7.5 ± 0.7 bc	19.3 ± 0.7 abc
200 mg/L YE	18.7 ± 1.7 bc	18.6 ± 1.5 bc	9.7 ± 1.2 b	21.2 ± 2.6 abc
50 mg/L SA	26.6 ± 1.3 a	31.4 ± 3.8 a	13.6 ± 1.6 a	28.3 ± 6.1 ab
100 mg/L SA	17.6 ± 0.6 cd	7.5 ± 1.4 efgh	6.9 ± 1.2 cde	22.7 ± 3.8 abc
200 mg/L SA	10.9 ± 0.5 e	3.6 ± 0.3 gh	4.4 ± 0.2 e	18.1 ± 3.9 bc
50 mg/L CdCl_2_	14.7 ± 1.5 d	16.5 ± 0.6 c	6.1 ± 1.1 cde	22.2 ± 3.3 abc
100 mg/L CdCl_2_	6.3 ± 1.6 f	8.3 ± 1.1 efg	0.0 ± 0.0 f	22.3 ± 1.4 abc
200 mg/L CdCl_2_	1.9 ± 0.2 g	2.5 ± 0.4 h	0.0 ± 0.0 f	18.5 ± 3.2 bc
50 mg/L AgNO_3_	7.1 ± 1.5 f	11.1 ± 1.0 de	5.3 ± 1.1 cde	21.6 ± 4.9 abc
100 mg/L AgNO_3_	2.1 ± 0.2 g	7.1 ± 1.0 efgh	4.5 ± 0.9 de	14.2 ± 0.9 c
200 mg/L AgNO_3_	2.0 ± 0.4 g	5.1 ± 0.7 gh	0.0 ± 0.0 f	14.4 ± 1.8 c

* Data were collected at the 11th week of culture. Values represent the mean ± SE. Mean values following the same letter within columns are not significantly different, according to Duncan’s multiple range (*p* ≤ 0.05) test.
